# Effectiveness of a multicomponent perioperative preparation programme incorporating culturally familiar clown-themed components for alleviating preoperative anxiety in preschool children

**DOI:** 10.3389/fped.2026.1776699

**Published:** 2026-04-10

**Authors:** Dandan Luo, Manman Luo, Zhaojun Qiu, Ming Yuan, Xin Huang, Tianyuan Luo, Yu Li

**Affiliations:** 1 Operating Room, Affiliated Hospital of Zunyi Medical University, Zunyi, Guizhou, China; 2 Department of Pediatrics, Affiliated Hospital of Zunyi Medical University, Zunyi, Guizhou, China; 3 Department of Anesthesiology, Affiliated Hospital of Zunyi Medical University, Zunyi, Guizhou, China

**Keywords:** anesthesia compliance, clown-themed, culturally familiar, pain behavior, preoperative anxiety, preschool children, surgery

## Abstract

**Objective:**

To develop a multicomponent perioperative preparation programme incorporating culturally familiar clown-themed components based on the distraction model, evaluate its efficacy in alleviating preoperative anxiety in preschool children, and provide an evidence-based non-pharmacological intervention strategy for the clinical management of preoperative anxiety in this population.

**Methods:**

The multicomponent perioperative preparation programme incorporating culturally familiar clown-themed components program was constructed via literature review, the Delphi method, and a pre-experiment. A total of 128 preschool children undergoing elective surgery at the Affiliated Hospital of Zunyi Medical University from November 2024 to July 2025 were randomly assigned to either a control group (receiving routine nursing care) or an experimental group (receiving routine nursing care combined with multicomponent perioperative preparation programme incorporating culturally familiar clown-themed components). The Simplified Modified Yale Preoperative Anxiety Scale (m-YPAS-SF), Modified Behavioral Pain Scale (MBPS), Induction Compliance Checklist (ICC), and electrocardiographic monitoring were utilized to assess anxiety levels, anesthetic procedural pain, anesthesia compliance, and heart rate at three time points: pre-intervention (T0), post-intervention (T1), and during anesthesia induction (T2).

**Results:**

No statistically significant differences were observed in general characteristics (gender, age, surgical approach, preference for culturally familiar cartoon images) between the two groups (*P* > 0.05). At T1 and T2, the experimental group exhibited significantly lower anxiety scores than the control group (*P* < 0.05). During anesthesia induction, the experimental group had a lower MBPS score and a superior ICC score compared with the control group (*P* < 0.05). Additionally, the experimental group demonstrated significantly lower heart rates at T1 and T2 than the control group (*P* < 0.05).

**Conclusion:**

The multicomponent perioperative preparation programme incorporating culturally familiar clown-themed components effectively mitigates preoperative anxiety, reduces anesthetic procedural pain, and improves anesthesia compliance in preschool children in this single-center study, which suggests its potential clinical applicability and warrants further validation for wider promotion.

## 1 Introduction

Preoperative anxiety is a prevalent psychological stress response in preschool children during the perioperative period, with an incidence of 40%–60% (1). This psychological stress not only predisposes children to anesthesia induction resistance, exacerbated postoperative pain, and delirium but also amplifies parental anxiety, thereby disrupting the smooth progression of clinical workflows (2). Traditional pharmacological interventions are associated with the risk of postoperative cognitive impairment and fail to address anxiety proactively (3). As a non-pharmacological intervention, clown care alleviates children's anxiety through humorous interactions that divert attention, and its efficacy has been validated in both outpatient and inpatient settings (4–6). However, most existing studies employ standardized foreign clown images, lacking localized and personalized adaptations, and the evidence supporting its skill use and goal for preoperative anxiety remains limited (7). Domestic research has demonstrated that clown care can reduce pain during intravenous indwelling needle puncture and outpatient dressing changes, but few studies have focused on preoperative anxiety alleviation (8). Furthermore, conventional clown care adopts a one-size-fits-all approach with uniform clown images, neglecting cultural adaptation and individual preferences. Notably, Chinese preschool children are more familiar with culturally rooted cartoon characters (e.g., GG Bond, Ultraman, Super Wings) than foreign-style clowns, and exposure to unfamiliar clown images may even induce cognitive conflict and heighten fear.

To address these gaps, the present study developed a multicomponent perioperative preparation programme incorporating culturally familiar clown-themed components by integrating Chinese cultural context and the psychological characteristics of preschool children. The primary objective was to evaluate whether this culturally tailored and individualized intervention could more effectively alleviate preoperative anxiety in preschool children compared with routine nursing care, while concurrently reducing anesthetic procedural pain and enhancing anesthesia compliance. Ultimately, this study aims to provide a feasible and humanistic non-pharmacological intervention strategy for clinical surgical nursing.

## 2 Materials and methods

### 2.1 Study design

This was a single-center randomized controlled trial conducted at the Affiliated Hospital of Zunyi Medical University. The study protocol was approved by the hospital ethics committee (Ethics Approval No.: KLL-2024-563) and was carried out in accordance with the Declaration of Helsinki. Informed consent was obtained from the guardians of all participating children, and children's consent was expressed by choosing their favorite clown toys. This study was designed and reported in accordance with the CONSORT 2010 Statement for randomized controlled trials, and a CONSORT flow diagram was provided to report the participant recruitment and follow-up process in detail.

### 2.2 Participants

A total of 128 preschool children undergoing elective surgery at the Affiliated Hospital of Zunyi Medical University between November 2024 and July 2025 were enrolled in this study.

Inclusion Criteria.

1. Aged 3–6 years old;
2. Undergoing elective surgery for the first time;
3. Scheduled for general anesthesia;
4. Guardians voluntarily participate in the study and sign the informed consent form;
5. Children can express their preferences through simple language or actions.

Exclusion Criteria.

(1) Children with cognitive and emotional disorders (e.g., autism, attention deficit hyperactivity disorder);
(2) Complicated with other serious diseases (e.g., heart disease, liver and kidney dysfunction);
(3) Unwillingness to cooperate with the study procedures (e.g., refusing to wear electrocardiographic monitoring, refusing to interact with volunteers);
(4) Children with a history of allergy to cartoon character costumes or related materials.

### 2.3 Routine nursing care protocol (control group)

All children in the control group received standardized routine pediatric perioperative nursing care in accordance with the clinical nursing guidelines for pediatric elective surgery of the Affiliated Hospital of Zunyi Medical University. No multicomponent perioperative preparation programme incorporating culturally familiar clown-themed components interventions, gamified interactions, or personalized reward mechanisms were implemented for the control group. The specific implementation contents and operational standards of routine nursing care were as follows:

(1) Preoperative health education and communication: The pediatric nurses provided verbal explanations of the elective surgery and general anesthesia process to the children's guardians in the ward, including the basic procedures of anesthesia induction, intraoperative care and postoperative recovery, and answered the guardians' questions about the operation and anesthesia. For preschool children, simple verbal comfort was given (e.g., “It will be over soon, be brave”) without age-appropriate gamified explanation or medical procedure familiarization.
(2) Anesthesia waiting room care: After the child was transferred to the anesthesia waiting room, the nurse maintained a quiet and comfortable environment, and allowed the guardians to accompany the child throughout the waiting period. Basic daily care was provided (e.g., adjusting the sitting posture, providing water), and no toys, cartoon videos or other distraction measures were specially prepared. No assessment of the child's emotional state, hobbies or cognitive development level was conducted, and no targeted psychological intervention was given for preoperative anxiety.
(3) Anesthesia induction room care: When the child entered the operating room for anesthesia induction, the circulating nurse and anesthesiologist performed the standard anesthesia induction procedure in accordance with the hospital's pediatric anesthesia operation specifications. Routine verbal encouragement was given during the operation (e.g., “Don't move, it will be fine soon”), without personalized praise, role-playing explanation of medical procedures or cartoon sticker pasting. The guardians were not allowed to enter the operating room during anesthesia induction in accordance with the hospital's infection control regulations.
(4) Post-anesthesia recovery care: After the completion of anesthesia induction and surgery, the child was transferred to the post-anesthesia care unit (PACU). The nurses monitored the child's vital signs (heart rate, blood pressure, oxygen saturation) in accordance with the standard process, and provided symptomatic nursing for discomfort such as pain and nausea. No agreed rewards were given to the child, and only routine inquiry about the subjective feelings was conducted when the child regained consciousness.

### 2.4 Sample size calculation

Using Gpower 3.1.9.2 software, a two-independent-sample *t*-test with a two-tailed alpha level of 0.05 was used for sample size calculation. Referring to similar studies (8), a medium effect size (*d* = 0.5) was assumed, with a statistical power (1 − *β*) of 0.8 and an equal sample size ratio (1:1) between the two groups. The calculated total sample size was 128 cases, with 64 cases in each group. The control group and the experimental group adopted the identical standardized pediatric anesthesia and analgesia protocols throughout the perioperative period, including the type and dosage of anesthetic drugs, the method of anesthesia induction, and the postoperative analgesic plan. The only difference between the two groups was whether the multicomponent perioperative preparation programme incorporating culturally familiar clown-themed components intervention was implemented on the basis of routine nursing care, which ensured the comparability of the two groups and eliminated the interference of anesthesia and analgesia measures on the research results.

### 2.5 Randomization and blinding

Simple randomization was used for participant allocation at a 1:1 ratio for the control and experimental groups. The random sequence was generated via the RAND function in Microsoft Excel 2019 by an independent biostatistician who was not involved in participant enrollment, intervention delivery, outcome assessment or data analysis. Sealed opaque envelope technique was applied for allocation concealment: the biostatistician serially numbered the random allocation results, sealed each result in a numbered opaque envelope, and stored the envelopes in a locked cabinet accessible only to the dedicated research nurse for enrollment. Upon enrolling a qualified preschool child, the research nurse sequentially opened the envelope with the smallest serial number and assigned the child to the corresponding group per the enclosed result, ensuring unpredictable group allocation and avoiding selection bias.

Due to the distinct visual and interactive features of the multicomponent perioperative preparation programme incorporating culturally familiar clown-themed components intervention, blinding was not feasible for children, their guardians, and intervention implementers (volunteers, circulating nurses, anesthesiologists). However, outcome assessors (responsible for m-YPAS-SF, MBPS and ICC scoring) and data analysts were strictly blinded to group allocation throughout the study. Blinding was maintained by separating the intervention and assessment teams, with only de-identified participant numbers (excluding group information) used for outcome measurement, data entry and statistical analysis. The blind was unbroken until all statistical analyses were completed.

The experimental group received the above-mentioned standardized routine nursing care combined with the multicomponent perioperative preparation programme incorporating culturally familiar clown-themed components intervention (detailed in 1.6.4), while the control group only received the standardized routine nursing care as specified in 1.3, with no additional intervention measures implemented. This ensured that the only variable between the two groups was the multicomponent perioperative preparation programme incorporating culturally familiar clown-themed components intervention.

### 2.6 Development of the culturally familiar clown-themed care program

This program is a multicomponent perioperative preparation programme incorporating culturally familiar clown-themed components (the innovative focus of this study) and general child-friendly perioperative preparation strategies (routine supportive measures for pediatric perioperative care). The following sections detail the development process and specific implementation of the program, with clear differentiation between the two types of components in the operational protocol.

#### 2.6.1 Literature review

**Figure 1:**
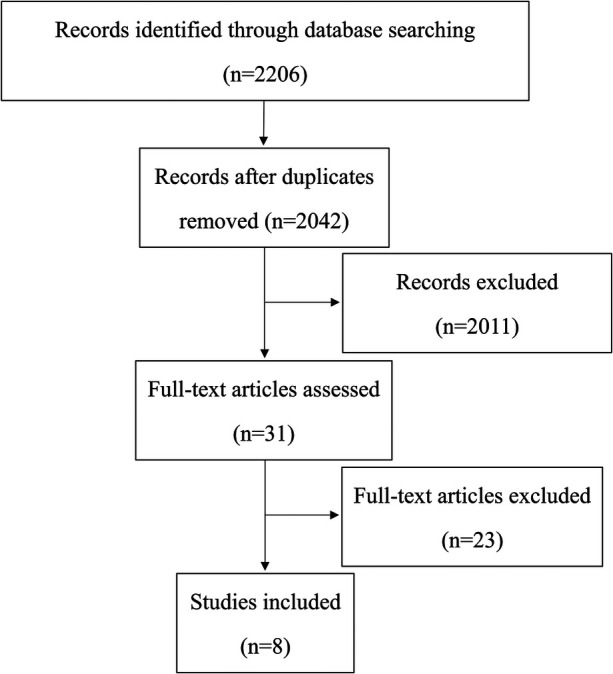
Framework development process of the culturally familiar clown-themed intervention programme.

Databases including CNKI and PubMed were systematically searched, and 8 randomized controlled trials on clown care for children aged 3–6 years were included to establish the initial program framework. (Figure 1).

#### 2.6.2 Delphi method

Twelve experts (specializing in pediatric nursing, anesthesiology, psychology, etc., with ≥10 years of clinical experience and an associate senior or higher professional title) were invited to conduct two rounds of expert consultation. The expert authority coefficient ranged from 0.871 to 0.900, and the effective response rate was 80%–100%. The program was revised based on expert feedback to ensure cultural adaptability and individualization.

#### 2.6.3 Core program design

Centered on cultural adaptation and personalization, the program incorporated cartoon characters familiar to Chinese preschool children (e.g., GG Bond, Ultraman, Super Wings, Briar/Bramble, Logger Vick, Paw Patrol) based on pre-survey data regarding children's preferences. Through gamified interactions, magical storytelling, and reward mechanisms, the program aimed to reduce children's fear of the hospital environment and medical procedures, thereby alleviating preoperative anxiety.

#### 2.6.4 Specific implementation of the program

This intervention is a **multicomponent perioperative preparation programme** consisting of culturally familiar clown-themed components and general child-friendly perioperative strategies. No single component can be isolated as the sole effective factor in this trial. The multicomponent perioperative preparation programme incorporating culturally familiar clown-themed components (C-CC) are the key design of this study, which are tailored to the cultural background and psychological characteristics of Chinese preschool children—mainly including the impersonation of culturally familiar cartoon/clown characters, magical storytelling with clown themes, personalized challenge games and reward mechanisms with clown elements. These components are the specific measures to address the deficiencies of existing clown care (lack of cultural adaptation and individualization). The general child-friendly perioperative preparation strategies (G-PS) are routine supportive nursing measures widely used in pediatric perioperative care, including operating room familiarization via video, gentle comfort and positive encouragement from medical staff, and subjective feedback collection. The two types of components are combined to form the multicomponent intervention program in this study, and the specific implementation is carried out in accordance with the stages of perioperative anesthesia management. The specific implementation of the intervention is shown in Table 1.

**Table 1:** Culturally familiar personalized clown care intervention protocol by stage and location.

Intervention stage	Intervention location	Intervention measures	Implementer	Intervention component type
1. Pre-induction Preparation	Anesthesia Waiting Room	① Based on pre-survey data, wear costumes of children's favorite culturally familiar cartoon characters (e.g., GG Bond, Ultraman) and imitate the characters' tone to attract children's attention; introduce the intervention purpose and methods to parents to obtain consent and encourage parental participation in interaction.	Trained Volunteers	C-CC
		② Take the initiative to communicate with children to understand their habits and hobbies, assess emotional state and cognitive development, and establish a trusting relationship with children in the cartoon character role.	Trained Volunteers	C-CC
		③ Play the operating room tour video to help children familiarize themselves with relevant procedures and eliminate fear of mask placement, electrocardiographic monitoring, and indwelling needle puncture during anesthesia induction.	Trained Volunteers	G-PS
		④ Narrate the “magic story” of the clown doctor: the oxygen mask is a magic tool for falling asleep, the fruity scent of anesthesia is the magic spell, and the clown doctor will defeat the “body monsters” during the child's sleep and leave a “little warrior mark” (puncture/indwelling needle site).	Trained Volunteers	C-CC
		⑤ Launch the anesthesia induction challenge game with the clown doctor/nurse; the child who completes the challenge will be awarded a “little warrior medal” to reduce the sense of strangeness to medical procedures.	Trained Volunteers	C-CC
2. Anesthesia Induction	Operating Room	① The circulating nurse gently touches the child's hand to comfort and encourage them, and pastes the child's favorite cartoon stickers on the arm.	Circulating Nurse	C-CC
		② Make a verbal agreement with the child that a predetermined reward will be given after successful cooperation with anesthesia induction.	Circulating Nurse + Trained Volunteers	C-CC
		③ The anesthesiologist and circulating nurse give intermittent praise and encouragement, and explain the injection/procedure in age-appropriate performance (e.g., “injection is like a mosquito bite, the liquid is magic to protect the body”).	Anesthesiologist + Circulating Nurse	G-PS
3. Post-anesthesia Recovery	Post-Anesthesia Care Unit	① Give the child the agreed reward as promised after anesthesia recovery.	Circulating Nurse	C-CC
		② Inquire about the child's subjective feelings and collect feedback to analyze the implementation effect of the intervention and areas for improvement.	Research Nurse	G-PS

C-CC, Culturally familiar clown-themed core components; G-PS, General child-friendly perioperative preparation strategies. This intervention represents a multicomponent bundle; the independent effect of clown-themed or culturally familiar components was not isolated in this study.

### 2.7 Evaluation indicators

Primary and secondary outcome indicators were pre-specified for this study in accordance with the research objective of alleviating preoperative anxiety in preschool children undergoing elective surgery. The primary outcome indicator was the score of the Simplified Modified Yale Preoperative Anxiety Scale (m-YPAS-SF) at the time of anesthesia induction (T2). Secondary outcome indicators included physiological and behavioral indicators: (1) Physiological indicator: heart rate at post-intervention (T1) and anesthesia induction (T2); (2) Behavioral indicators: Modified Behavioral Pain Scale (MBPS) score and Induction Compliance Checklist (ICC) score at anesthesia induction, as well as m-YPAS-SF scores at pre-intervention (T0) and post-intervention (T1).

Three measurement time points were precisely defined with unified setting and environmental conditions, and all outcome assessments were conducted in strict accordance with the following time nodes and scenarios: T0 (pre-intervention): Upon the child's admission to the anesthesia waiting room, no intervention measures were implemented, the child's guardian was present, and no pre-anesthetic drugs were administered; T1 (post-intervention): Immediately after the completion of all intervention measures (about 20–30 min), the child remained in the anesthesia waiting room with the guardian present; T2 (during anesthesia induction): At the initiation of anesthesia induction operation after the child entered the operating room, the guardian was not allowed to enter the operating room in accordance with infection control regulations, and no anesthetic induction drugs were used before assessment.

All behavioral outcome assessments (m-YPAS-SF, MBPS, ICC) were completed by two trained postgraduate students majoring in pediatric nursing who had no involvement in the intervention implementation. The assessors received standardized professional training on the use of the three scales, including scale scoring criteria, observation skills and judgment standards. A pre-experiment was conducted after training, and the inter-rater reliability was tested using the intraclass correlation coefficient (ICC). The results showed that the ICC of all scale assessments was > 0.85, indicating good consistency of the assessment results. Heart rate was measured by the on-duty circulating nurses using a standard electrocardiographic monitor, and the measurement operation was standardized in accordance with the hospital's nursing operation specifications.

Blinding was strictly implemented for outcome assessment and data analysis to reduce measurement bias. Outcome assessors and data analysts were kept blinded to the participants' group allocation throughout the entire study process. The blinding measures included: (1) Separation of the intervention implementation team and the outcome assessment team, with no information exchange between the two teams; (2) Only de-identified participant numbers were used for outcome assessment, data entry and statistical analysis, without any group identification information; (3) The random allocation results were kept confidential by the independent biostatistician until all statistical analyses were completed. Given the obvious visual and interactive characteristics of the intervention, it was impossible to blind the intervention implementers, children and their guardians, and this limitation was fully considered in the study design.

#### 2.7.1 General data questionnaire

A self-designed general data questionnaire was used to investigate the surgical children, including gender, age, surgical method, anesthesia method, and the child's favorite clown image. In addition to the basic demographic data, the questionnaire also collected clinical data including ASA physical status classification (assessed by attending anesthesiologists), surgical invasiveness (divided into minimally invasive and open surgery according to surgical procedures), and pre-anesthetic medication use (recorded by nursing staff). Family-related variables included parental presence expectation (collected by verbal inquiry) and parental preoperative anxiety score [assessed by the Self-Rating Anxiety Scale (SAS), a validated scale with good reliability and validity for measuring adult anxiety levels].

#### 2.7.2 Anxiety level

Differences in anxiety between the two groups were evaluated by the modified Yale Preoperative Anxiety Scale-Short Form (m-YPAS-SF) (9). It includes 18 items, divided into 4 dimensions: “Activity”, “Language Ability”, “Emotional Expression Ability”, and “Arousal State”. The scoring method is to assign 1–4 points or 1–6 points according to the number of items in different parts, and convert them into a 100-point scale. The specific conversion method is: actual score of each part = (total score of items in each part ÷ number of items) × (100 ÷ number of parts). The sum of the actual scores of each part is the total score, with a total score range of 23–100 points. A higher score indicates a higher level of anxiety in the child.

#### 2.7.3 Pain level

The Modified Behavioral Pain Scale (MBPS) was used to assess children's procedural pain, which is a validated scale suitable for evaluating pain in preschool children during invasive medical procedures such as venipuncture, indwelling needle puncture, and anesthesia induction-related operations. Two time points of MBPS assessment were set in this study: the first was the baseline assessment at T0 (pre-intervention, in the anesthesia waiting room with no invasive procedures implemented), and the second was the formal pain assessment at T2 (during anesthesia induction, at the time of invasive operations such as venipuncture and mask ventilation). All observations, evaluations and scoring were completed by two trained postgraduate students majoring in pediatric nursing with good inter-rater reliability (ICC > 0.85). The MBPS score consists of three dimensions with a total score range of 0–9 points, and the scoring criteria are as follows: 1) Facial expression: positive/pleasant expression (e.g., smiling) = 0 points, neutral expression = 1 point, mild discomfort (e.g., grimacing) = 2 points, obvious distress (e.g., frowning, tightly closing eyes) = 3 points; 2) Crying behavior: no crying = 0 points, groaning/whimpering = 1 point, soft crying = 2 points, loud screaming/crying = 3 points; 3) Body movement: relaxed/quiet, voluntary exposure of the operative site (e.g., upper arm) = 0 points, mild tension/arching wriggling, stiff limbs = 1 point, limb withdrawal/contracting to avoid pain = 2 points, severe restlessness or complete body stiffness = 3 points. The total MBPS score is the sum of the scores of the three dimensions, with a higher total score indicating a more severe level of procedural pain perceived by the child.

#### 2.7.4 Anesthesia induction compliance

The ICC (10) includes 1 item, which is used to assess the negative behavioral performance of children during anesthesia induction. If the child has no negative behaviors during the induction process, that is, the anesthesia induction is smooth, it is recorded as 0 points; each negative behavior is recorded as 1 point, and the sum of these scores is the total score during the induction period, with a maximum total score of 10 points. Among them, 0 points indicate extremely high compliance of the child and a very smooth induction process; 1–5 points indicate a relatively smooth induction process for the child; >5 points indicate poor compliance of the child. A higher score indicates lower compliance of the child.

#### 2.7.5 Physiological indicator (heart rate)

Heart rate was measured using an electrocardiographic monitor at T0, T1, and T2 to reflect physiological responses to anxiety and stress. Three consecutive measurements were taken at each time point with an interval of 1 min, and the average value was recorded as the final heart rate data to ensure the accuracy of the measurement results.

### 2.8 Ethical considerations

This study strictly followed ethical principles and obtained the approval of the hospital ethics committee before formal implementation (Ethics Approval No.: KLL-2024-563). This study adhered to the principles of voluntariness, confidentiality, and benefit. The purpose of this survey was explained to the children and their families. Due to the limited cognitive ability of the children, the research process was explained through simple gamified language. The children's consent was expressed by letting them choose their favorite clown toys, and the informed consent forms were signed by the children and their families.

### 2.9 Statistical methods

All statistical analyses were performed using IBM SPSS 29.0 software. Continuous variables were expressed as mean ± standard deviation ($\bar{x}\pm s$), and ordinal categorical variables were presented as median (interquartile range) M (P25,P75). Count data were described by frequency and percentage (*n*,%).

For repeated measurement indicators [Simplified Modified Yale Preoperative Anxiety Scale (m-YPAS-SF) score and heart rate (HR) at T0, T1, T2], a linear mixed-effects model (LMM) was used to analyze the main effects of group (experimental vs. control) and time (T0/T1/T2), as well as their interaction effect (group × time). Bonferroni *post-hoc* test was conducted for pairwise comparisons when a significant interaction effect was identified. The results were reported with F value, degrees of freedom (df), *P* value, Cohen's d effect size and 95% confidence interval (95%CI) for mean differences to quantify the clinical significance of intervention effects.

For ordinal categorical variables [Modified Behavioral Pain Scale (MBPS) score and Induction Compliance Checklist (ICC) score], Mann–Whitney *U*-test was adopted for inter-group comparison due to the non-normal distribution. The results were reported with Z-value, *P*-value, median difference and its 95%CI. All statistical analyses were conducted based on the intention-to-treat (ITT) principle, with all randomized participants included in the analysis according to their original group allocation.

All statistical tests were two-sided, and a *P*-value < 0.05 was considered statistically significant. The Bonferroni correction was applied for multiple pairwise comparisons to control the type Ⅰ error rate.

## 3 Results

All results were reported in the order of primary and secondary outcome indicators pre-specified in the study design. Baseline characteristics of the two groups were first compared to verify the comparability of the samples, followed by the analysis of the primary outcome indicator, and then the statistical analysis of each secondary outcome indicator.

### 3.1 Baseline characteristics of participants

**Figure 2:**
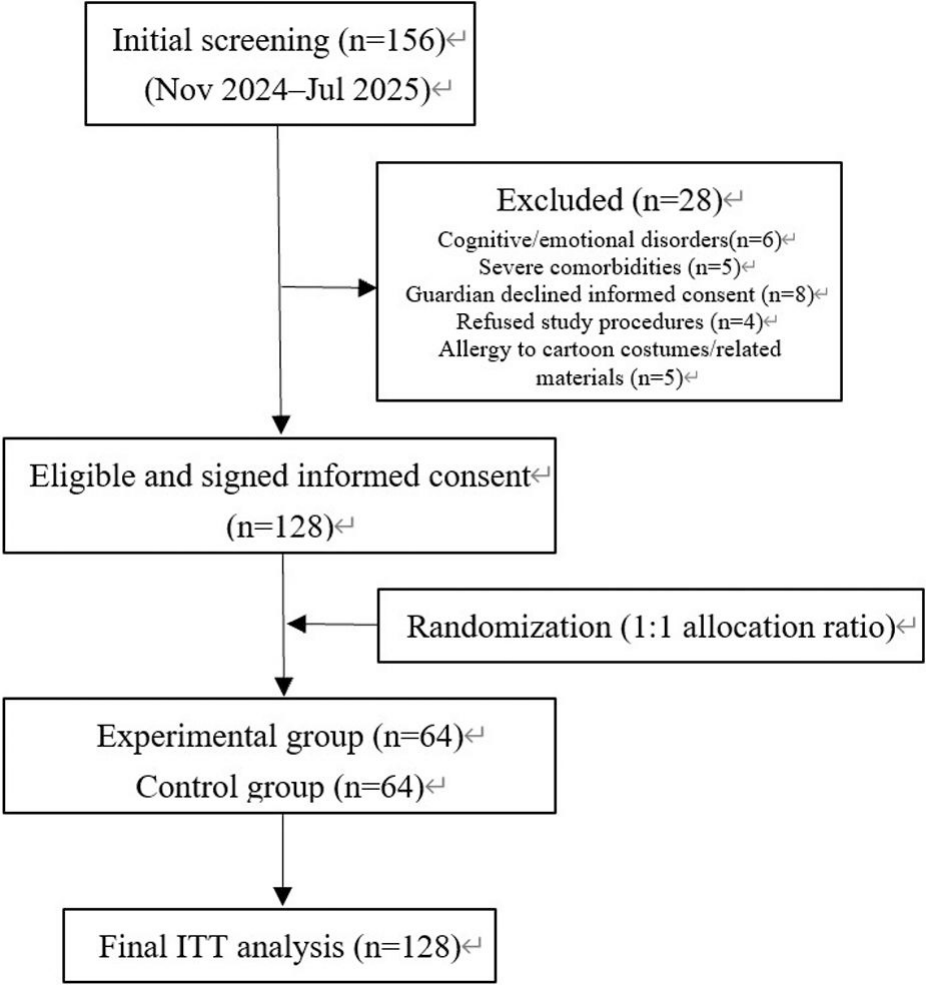
CONSORT flow diagram of participant recruitment, randomization and follow-up in the study.

This study strictly followed the CONSORT 2010 Statement for the reporting of randomized controlled trials. The flow of preschool children participating in the study from screening to final statistical analysis is as follows: a total of 156 preschool children who underwent elective surgery at the Affiliated Hospital of Zunyi Medical University were initially screened between November 2024 and July 2025. Among them, 28 children were excluded for the following reasons: 6 children with cognitive and emotional disorders (attention deficit hyperactivity disorder), 5 children with complicated serious systemic diseases (liver and kidney dysfunction), 8 children whose guardians refused to sign the informed consent form, 4 children who were unwilling to cooperate with the study procedures (refusing electrocardiographic monitoring, rejecting interaction with volunteers), and 5 children with a history of allergy to cartoon character costumes or related materials. Finally, a total of 128 eligible children were enrolled in the study and randomly assigned to the control group (*n* = 64) and the experimental group (*n* = 64) at a 1:1 allocation ratio. No children were lost to follow-up or excluded after randomization, and all 128 enrolled children completed the study and were included in the final intention-to-treat (ITT) analysis (Figure 2).

In addition to gender, age, surgical approach and preference for culturally familiar cartoon images, key clinical and family-related variables including American Society of Anesthesiologists (ASA) physical status classification, surgical invasiveness, pre-anesthetic medication use, parental presence expectation and parental preoperative anxiety score were also collected for baseline comparison. No statistically significant differences were observed in any of the baseline variables between the two groups (*P* > 0.05), indicating that the baseline characteristics were well balanced and comparable between the two groups (Table 2).

**Table 2:** Comparison of general data between the Two groups of surgical children ($\bar{x}\pm s/n$).

Variables	Subgroups	Control group (*n* = 64)	Experimental group (*n* = 64)	*t*/*χ*^2^ Value	*P* Value
Age (years)	–	4.53 ± 1.07	4.89 ± 1.01	1.956	0.053
Gender (*n*)	Male	49	42	1.863^a^	0.172
	Female	15	22		
ASA physical status classification (*n*)	Ⅰ	58	60	0.512^a^	0.774
	Ⅱ	6	4		
Surgical invasiveness (*n*)	Minimally invasive surgery	60	62	0.418^a^	0.518
	Open surgery	4	2		
Pre-anesthetic medication use (*n*)	Yes	11	9	0.347^a^	0.556
	No	53	55		
Surgical Method (*n*)	Laparoscopic High Ligation of Hernia Sac	18	9	5.310^a^	0.070
	Laparoscopic High Ligation of Processus Vaginalis	16	13		
	Endoscopic Adenotonsillectomy	30	42		
Parental preoperative anxiety score (points)	–	35.26 ± 5.18	34.89 ± 4.92	0.395	0.693
Parental presence expectation (*n*)	Yes	59	61	0.421^a^	0.516
	No	5	3		
Clown Image (*n*)	Mr. Clown	21	19	7.054^a^	0.136
	GG Bond	7	10		
	Ultraman	10	12		
	Super Wings	4	11		
	Briar/Bramble	9	5		
	Logger Vick	8	4		
	Paw Patrol	5	3		

a Indicates χ^2^ value.

### 3.2 Comparison of preoperative anxiety levels between the two groups

**Figure 3:**
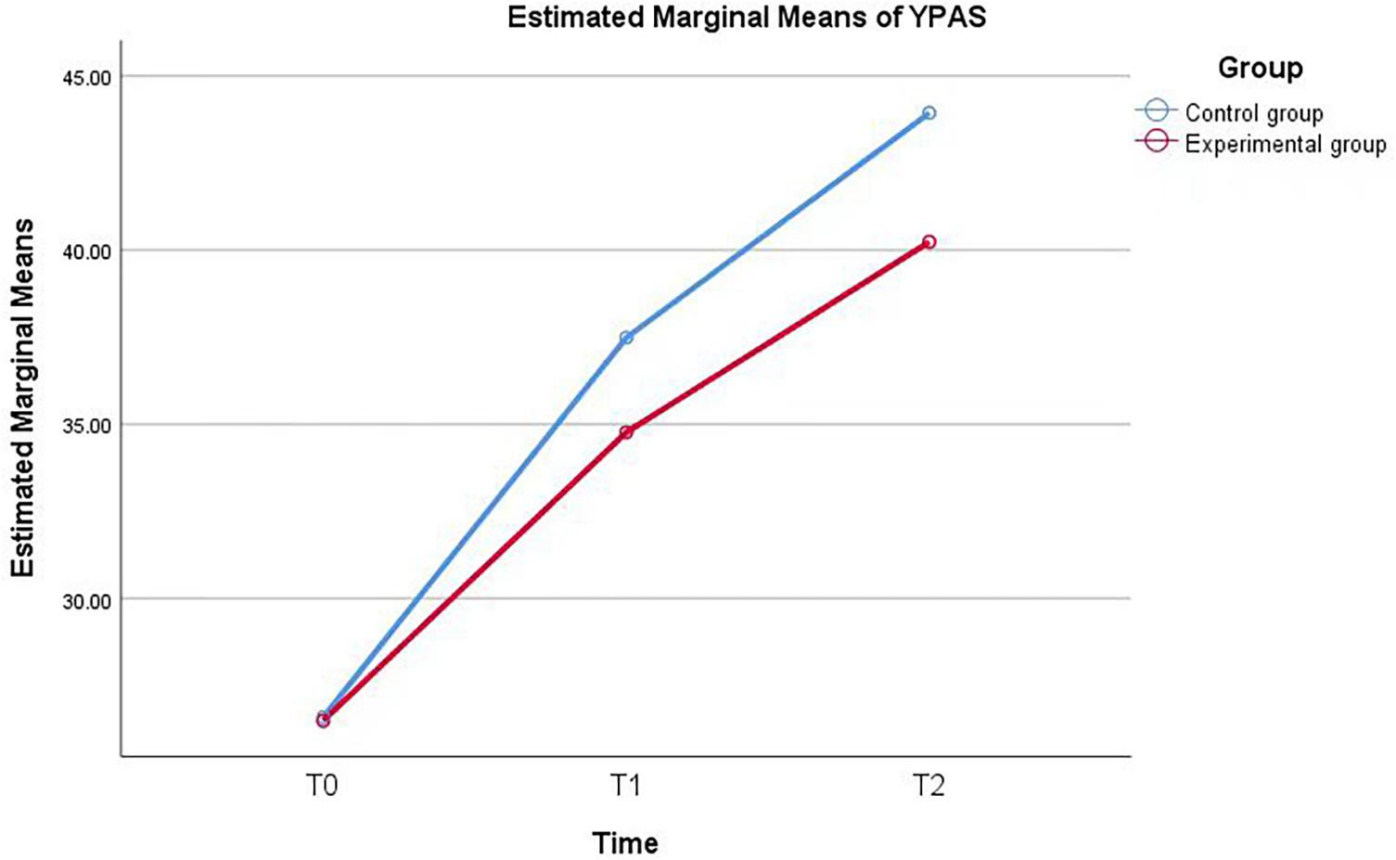
Estimated marginal means of m-YPAS-SF anxiety scores in the two groups over time. The blue line represents the control group, and the red line represents the experimental group. A significant group ×  time interaction effect was observed (*F* = 4.576, df = 2, *P* = 0.011).

A linear mixed-effects model revealed significant main effects of group and time, as well as a significant group × time interaction effect. At T0, the two groups showed comparable m-YPAS-SF scores. At T1, the experimental group had significantly lower anxiety scores than the control group, and this difference was further enlarged at T2, indicating a moderate-to-large intervention effect (Table 3, Figure 3).

**Table 3:** Comparison of m-YPAS-SF anxiety scores between the two groups at different time points ($\bar{x}\pm s$, points).

Groups	*n*	T0	T1	T2
Experimental Group	64	26.48 ± 3.69	34.77 ± 4.34^a^	40.23 ± 5.70^a^
Control Group	64	26.59 ± 2.92	37.48 ± 5.29	43.94 ± 6.61
Mean difference	–	0.10	2.72	3.70
95%CI	–	(−1.61, 1.81)	(1.01, 4.43)	(1.99, 5.41)
Cohen's d	-	0.03	0.56	0.60

Linear mixed-effects model results: (1) Group main effect: *F* = 18.767, df = 1, *P* < 0.001. (2) Time main effect: *F* = 325.597, df = 2, *P* < 0.001. (3) Group × time interaction: *F* = 4.576, df = 2, *P* = 0.011.

a *P* < 0.05 vs. control group.

### 3.3 Comparison of MBPS scores between the Two groups

The MBPS score was analyzed using the Mann–Whitney *U*-test. Baseline MBPS scores at T0 were comparable between the two groups (*P* > 0.05). At T2, the experimental group exhibited significantly lower pain scores than the control group with a median difference of −1.14, suggesting that the intervention effectively reduced children's procedural pain perception (Table 4).

**Table 4:** Comparison of MBPS pain scores between the two groups (M, P25, P75).

Groups	*n*	T2	Mann–Whitney U	*Z value*	*P value*	Median difference (95%CI)
Experimental Group	64	4.14 ± 2.05^a^	1,382.50	−3.207	0.001	−1.14 (−1.82, −0.46)
Control Group	64	5.28 ± 1.83	–	–	–	–

MBPS was analyzed by Mann–Whitney *U*-test.

a *P* < 0.05 vs. control.

### 3.4 Comparison of induction compliance between the two groups

The ICC score was analyzed using the Mann–Whitney *U*-test. The experimental group had significantly lower ICC scores than the control group at T2, with a median difference of −1.00, indicating better anesthesia induction compliance in the experimental group (Table 5).

**Table 5:** Comparison of ICC anesthesia induction compliance scores between the two groups (M, P25, P75).

Groups	*n*	T2	Mann–Whitney U	*Z value*	*P value*	Median difference (95%CI)
Experimental Group	64	2.34 ± 2.00^a^	1,351.00	−3.366	<0.001	−1.00 (−1.63, −0.37)
Control Group	64	3.34 ± 1.49	–	–	–	–

ICC was analyzed by Mann–Whitney *U*-test; lower score, better compliance.

a *P* < 0.05 vs. control.

### 3.5 Comparison of heart rate between the Two groups of children

**Figure 4:**
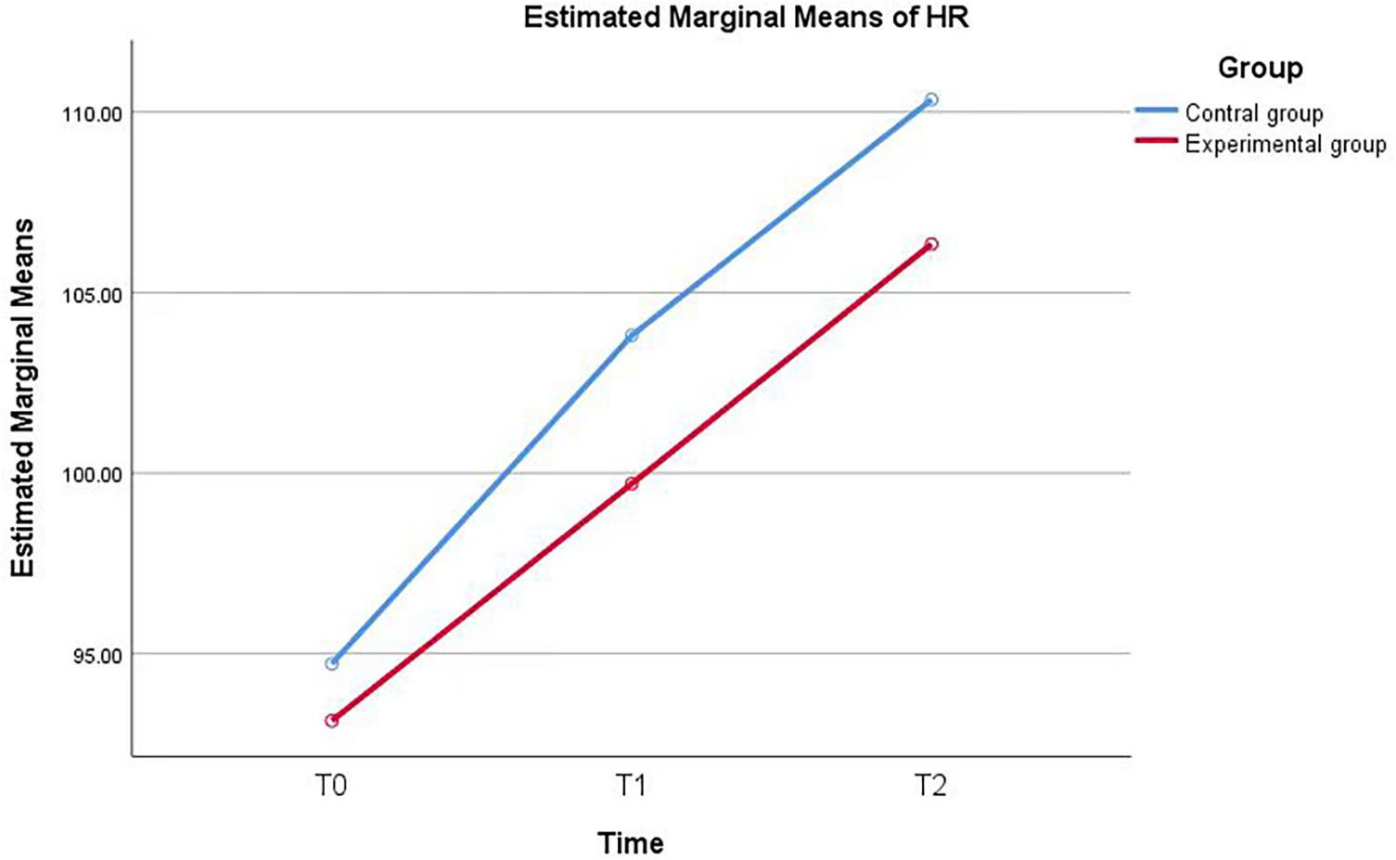
Estimated marginal means of heart rate (HR) in the two groups over time. Figure Legend: The blue solid line represents the control group (routine nursing care only), and the red solid line represents the experimental group (routine nursing care + culturally familiar personalized clown care intervention). No significant group × time interaction effect was observed (*F* = 1.002, df = 2, *P* = 0.368), indicating a consistent increasing trend in HR over time in both groups, while the experimental group maintained a lower HR level throughout the perioperative period.

A linear mixed-effects model showed significant main effects of group and time, but no significant group × time interaction effect. At T0, the two groups had comparable HR levels. The intervention group had significantly lower heart rates at T1 and T2, but the pattern of change over time did not differ significantly between groups (group ×  time interaction, *P* = 0.368). (Table 6, Figure 4).

**Table 6:** Comparison of heart rate between the two groups at different time points ($\bar{x}\pm s$, beats/min).

Groups	T0	T1	T2
Experimental Group	93.14 ± 7.76	99.70 ± 8.16^a^	106.34 ± 9.05^a^
Control Group	94.72 ± 7.69	103.81 ± 7.56	110.34 ± 8.01
Mean difference	1.58	4.11	4.00
95%CI	(−1.23, 4.39)	(1.30, 6.92)	(1.19, 6.81)
Cohen's d	0.20	0.52	0.47

Linear mixed-effects model results: (1) Group main effect: *F* = 15.309, df = 1, *P* < 0.001. (2) Time main effect: *F* = 101.929, df = 2, *P* < 0.001. (3) Group   ×   time interaction: *F* = 1.002, df = 2, *P* = 0.368.

a *P* < 0.05 vs. control group.

## 4 Discussion

### 4.1 Rationale for developing culturally familiar clown-themed care

Preoperative anxiety in preschool children is a complex psychological phenomenon caused by multiple factors, including unfamiliarity with the hospital environment, fear of pain, and separation from caregivers (11, 12). Traditional pharmacological interventions have limited effects and potential risks, while non-pharmacological interventions such as clown care have become an important direction for clinical intervention due to their safety and effectiveness (13). However, existing clown care models have obvious limitations: first, they lack cultural adaptation. Based on our preliminary pre-survey, foreign-style clowns may not resonate with Chinese preschool children and might potentially increase their fear, although no direct comparative data were collected in this study to verify this hypothesis; second, they lack individualization, and the “one-size-fits-all” intervention cannot meet the unique needs and preferences of different children (7, 8).

In this study, based on the distraction model, we developed a multicomponent perioperative preparation programme incorporating culturally familiar clown-themed components intervention. The key design elements integrated in this study include: (1) Cultural adaptation: Integrating cartoon characters familiar to Chinese preschool children (e.g., GG Bond, Ultraman) into the intervention, reducing cognitive dissonance and enhancing emotional resonance; (2) Individualization: Tailoring intervention content and forms according to children's preferences and cognitive development levels, enhancing their sense of participation and control; (3) Gamification: Adopting magical storytelling, challenge games, and reward mechanisms to divert children's attention from negative emotions to positive experiences, thereby alleviating anxiety. These innovative designs are all embodied in the culturally familiar C-CC of the intervention, and the G-PS are used as the basic support to ensure the completeness and smooth implementation of the perioperative nursing intervention.

### 4.2 Efficacy of the intervention in alleviating preoperative anxiety

The results of this study showed that at T1 and T2, the anxiety scores of the experimental group were significantly lower than those of the control group, indicating that culturally multicomponent perioperative preparation programme incorporating culturally familiar clown-themed components care can effectively alleviate preoperative anxiety in preschool children. This finding is consistent with the results of Dionigi et al. (13) and Markova et al. (14), who reported that clown-based interventions can reduce preoperative anxiety in children. Potential mechanisms hypothesized based on previous literature are as follows: (1) Familiar cartoon characters may reduce children's sense of strangeness to the intervention, potentially increasing their willingness to accept and participate in the interaction; (2) Gamified interactions and reward mechanisms may enhance children's sense of pleasure and achievement, which could help alleviate negative emotions such as anxiety and fear; (3) The establishment of a trusting relationship between volunteers and children may provide emotional support for children, assisting them in coping with preoperative stress related to medical procedures, and this may also contribute to improved parental satisfaction (though parental satisfaction was not a primary outcome in this study).

The intervention showed moderate-to-large effect sizes (Cohen's d ranging from 0.41 to 0.60) for reducing preoperative anxiety and heart rate, as well as improving pain and compliance. These effect sizes indicate that multicomponent perioperative preparation programme incorporating culturally familiar clown-themed components has not only statistical but also clinical significance in pediatric perioperative care.

### 4.3 Impact of the intervention on anesthetic procedural pain and anesthesia compliance

Pain during anesthesia induction is an important factor affecting children's cooperation and postoperative recovery. This study found that the MBPS score of the experimental group during anesthesia induction was significantly lower than that of the control group, indicating that the intervention could be associated with the reduction of children's perception of anesthetic procedural pain. Based on previous studies on non-pharmacological pain intervention in children, the multicomponent perioperative preparation programme incorporating culturally familiar clown-themed components intervention is hypothesized to induce positive emotions, which may promote endorphin secretion—endogenous analgesics—thereby reducing pain sensitivity (15), although no direct measurement of endorphin levels was performed in the present study to confirm this mediating pathway. In addition, the relaxed and pleasant intervention environment may also distract children's attention from pain, reducing their subjective perception of pain.

Anesthesia compliance is crucial for the smooth progress of anesthesia induction and surgery. The results showed that the ICC score of the experimental group was significantly lower than that of the control group, indicating that the intervention improved children's anesthesia compliance. This is consistent with the findings of Lopes-Júnior et al. (16), who reported that clown care can enhance children's compliance with medical procedures. The possible reasons are: (1) The intervention may reduces children's fear of anesthesia induction and related medical procedures, making them more willing to cooperate; (2) The reward mechanism and positive encouragement during the intervention enhance children's motivation to cooperate; (3) The participation of parents in the interaction provides emotional support for children, helping them reduce resistance during anesthesia induction, and ensured the smooth execution of clinical procedures.

### 4.4 Impact of the intervention on physiological stress responses

Heart rate is a sensitive physiological indicator reflecting anxiety and stress. This study found that heart rate was significantly lower in the intervention group at T1 and T2, but the temporal trend did not differ significantly between groups, suggesting a main effect of the intervention rather than a differential time-course effect in preschool children. Preschool children's cognitive ability is not fully developed, and they are prone to fear and anxiety when facing the unknown surgical environment and medical procedures, which leads to an elevated heart rate (11). The multicomponent perioperative preparation programme incorporating culturally familiar clown-themed components intervention alleviates children's anxiety through multiple ways, thereby reducing the activation of the sympathetic nervous system and lowering heart rate. Shimshi-Barash et al. (17) found that reducing preoperative anxiety in children can help improve sleep quality, shorten hospitalization time, and promote postoperative rehabilitation. These findings from prior research possible explanations was that the clown-themed intervention in the present study may have potential positive implications for children's physical and mental health, though short-term follow-up in this study did not assess these long-term outcomes.

## 5 Conclusion

The multicomponent perioperative preparation programme incorporating culturally familiar clown-themed components, as a non-pharmacological psychological intervention, was found to effectively alleviate preoperative anxiety, reduce anesthetic procedural pain, improve anesthesia compliance, and mitigate anxiety-induced physiological stress responses in preschool children in this single-center randomized controlled trial. This programme is designed in accordance with the psychological characteristics and cultural background of Chinese preschool children and shows good clinical feasibility and operability in our clinical setting. Its value for wider clinical promotion and application requires verification in multicenter studies with larger sample sizes and longer follow-up periods. This study provides a potential evidence-based reference for the clinical management of preoperative anxiety in preschool children and offers a feasible approach for the development of humanistic nursing in pediatric surgical care.

### 5.1 Limitations and future directions

This study has several limitations that warrant consideration. First, it was a single-center study with a relatively small sample size, and the follow-up period was short, which limits the generalizability of the results. Second, the study did not verify the hypothesized negative effect of foreign-style clown images on Chinese preschool children, nor did it conduct biological measurements (e.g., endorphin levels) to confirm the inferred mechanisms of the intervention's effect. Third, the intervention is a bundled multicomponent programme, and the present study was unable to isolate the independent effects of individual components (e.g., cultural adaptation, gamification) on the observed outcomes. Future research should expand the sample size, conduct multi-center studies, and implement long-term follow-up to verify the sustained efficacy of the intervention. Additionally, future studies could set up head-to-head comparison groups (e.g., foreign-style clown care vs. culturally familiar clown care) to validate the cultural fit of the intervention, conduct biological assays to explore its underlying mechanisms, and perform component analysis to identify the core effective elements of the programme. Further refinement for children of different age groups is also needed to enhance its individualization and clinical precision.

## Data Availability

The original contributions presented in the study are included in the article/Supplementary Material, further inquiries can be directed to the corresponding author.
